# (*R*)-3,4,5-Tride­oxy-5,6-didehydro-1,2-*O*-(2,2,2-trichloro­ethyl­idene)-α-d-gluco­furan­ose-6,3-carbolactone: a new derivative of α-chloralose

**DOI:** 10.1107/S1600536808026196

**Published:** 2008-08-20

**Authors:** Violeta Aburto-Luna, Rosa-Luisa Meza-León, Sylvain Bernès

**Affiliations:** a Centro de Investigación de la Facultad de Ciencias Químicas, Universidad Autónoma de Puebla, 72570 Puebla, Mexico; b Facultad de Ciencias Químicas, UANL, Guerrero y Progreso S/N, Col. Treviño, 64570 Monterrey, NL, Mexico

## Abstract

The title compound [systematic name: (*R*)-2-trichloro­methyl-3a,3b,7a,8a-tetra­hydro-5*H*-pyrano[2′,3′:4,5]furano[2,3-*d*][1,3]dioxol-5-one], C_9_H_7_Cl_3_O_5_, a triyclic system that contains a central α-d-furan­ose ring *cis*-fused with a dioxolane ring as well as a δ-lactone ring, exhibits a twisted conformation. The CCl_3_ group has an axial orientation. The furan­ose ring approximates an envelope conformation due to the α,β-unsaturated lactone functionality. The asymmetric unit contains two independent mol­ecules with almost identical geometries.

## Related literature

For background regarding α-chloralose and δ-lactones, see: Collins *et al.* (1983 ▶); Zosimo-Landolfo & Tronchet (1999 ▶); Wu *et al.* (1992 ▶).
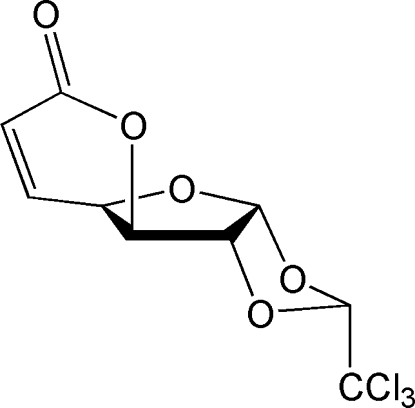


## Experimental

### 

#### Crystal data

C_9_H_7_Cl_3_O_5_
*M* *_r_* = 301.50
Orthorhombic, 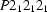

*a* = 9.129 (4) Å
*b* = 11.264 (4) Å
*c* = 23.156 (7) Å
*V* = 2381.1 (15) Å^3^
*Z* = 8
Mo *K*α radiation
μ = 0.77 mm^−1^
*T* = 298 (1) K
0.60 × 0.40 × 0.10 mm

#### Data collection

Siemens P4 diffractometer
Absorption correction: ψ scan (*XSCANS*; Siemens, 1996 ▶) *T*_min_ = 0.803, *T*_max_ = 0.926
6901 measured reflections
4735 independent reflections
3838 reflections with *I* > 2σ(*I*)
*R*_int_ = 0.044
3 standard reflections every 97 reflections intensity decay: 2%

#### Refinement

*R*[*F* ^2^ > 2σ(*F* ^2^)] = 0.044
*wR*(*F* ^2^) = 0.111
*S* = 1.06
4735 reflections
307 parameters
H-atom parameters constrained
Δρ_max_ = 0.34 e Å^−3^
Δρ_min_ = −0.35 e Å^−3^
Absolute structure: Flack (1983 ▶), 2010 Friedel pairs
Flack parameter: 0.04 (8)

### 

Data collection: *XSCANS* (Siemens, 1996 ▶); cell refinement: *XSCANS*; data reduction: *XSCANS*; program(s) used to solve structure: *SHELXS97* (Sheldrick, 2008 ▶); program(s) used to refine structure: *SHELXL97* (Sheldrick, 2008 ▶); molecular graphics: *Mercury* (Macrae *et al.*, 2006 ▶); software used to prepare material for publication: *SHELXL97*.

## Supplementary Material

Crystal structure: contains datablocks I, global. DOI: 10.1107/S1600536808026196/pv2095sup1.cif

Structure factors: contains datablocks I. DOI: 10.1107/S1600536808026196/pv2095Isup2.hkl

Additional supplementary materials:  crystallographic information; 3D view; checkCIF report

## Figures and Tables

**Fig. 1.**
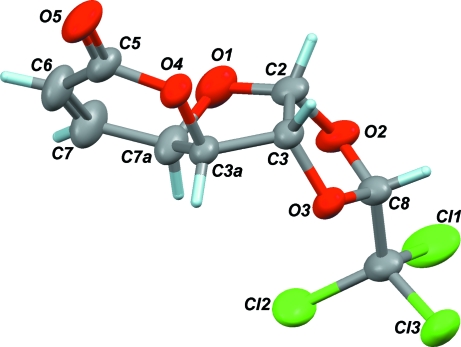
Structure of the first independent molecule. Displacement ellipsoids are shown at the 30% probability level.

**Fig. 2.**
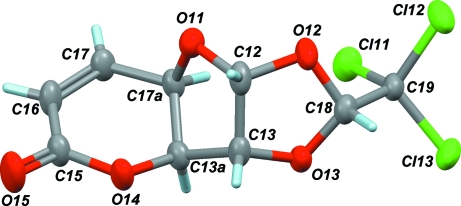
Structure of the second independent molecule. Displacement ellipsoids are shown at the 30% probability level.

**Fig. 3.**
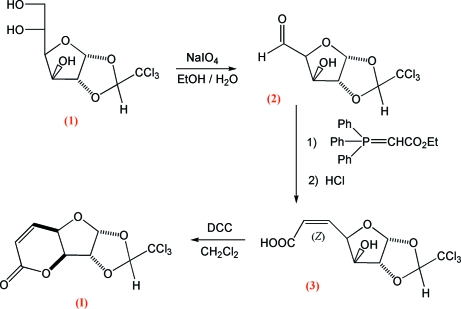
The synthesis of (I)

## supplementary crystallographic information

### Comment

α-Chloralose [1,2-*O*-(2,2,2-trichloroethylidene)-*α*-D-glucofuranose, (1) in Scheme 2] is an easily available carbohydrate derivative, bearing well studied biological properties. It is a mild hypnotic drug, which is currently used as an anesthetic in veterinary medicine, as a rodenticide, *etc*. It has been characterized as a molecule possessing potent *CNS* activity, and has been evaluated in human and animal models, for its therapeutic properties (Collins *et al.*, 1983). A number of α-chloralose derivatives have been prepared (*e.g*., Zosimo-Landolfo & Tronchet, 1999), since it is known that trichloroethylidene acetals are potential biologically active compounds.

On the other hand, δ-lactones are important flavor and aroma constituents found in many natural products. In some instances, δ-lactone derivatives have been shown to have anti-cancer and apoptosis inducing properties against various human tumors and animal cell lines (Wu *et al.*, 1992).

We have synthesized a compound combining both functionalities, (I), with the hope that this compound will also cumulate properties corresponding to each functionality. The starting material was α-chloralose, (1, scheme 2), which was first oxidized into an aldehyde, (2), and then transformed to the corresponding acrylic acid (3) *via* a Wittig reaction affording a pure *Z* isomer. Cyclization furnished the lactone (I).

The asymmetric unit of (I) contains two molecules (Figs. 1 and 2), with almost identical geometry. A fit between two independent molecules (non-H atoms) gives a r.m.s. deviation of 0.103 Å. The tricyclic system includes a central α-*D*-furanose ring approximating an envelope conformation, with C7a as flap atom (C17*a* in the other molecule). This ring is *cis*-fused with a dioxolane ring, which may be considered as twisted on O3 and C8 (O13 and C18, resp.). The CCl_3_ substituent has an axial orientation, as in α-chloralose. Finally, the α,β-unsaturated δ-lactone ring is *cis*-fused with the furanose, and displays a rigid envelope conformation, with a total puckering amplitude of 0.347 (4) Å [0.361 (4) Å for the second molecule]. Molecules are well separated in the crystal, and no significant intermolecular contacts are detected.

### Experimental

The synthesis of (I) is depicted in scheme 2. A solution of α-chloralose (5 g, 16.23 mmol) in ethanol (60 ml) was mixed with a solution of NaIO_4_ (3.47 g, 16.23 mmol) in H_2_O (6 ml). After stirring this solution at 298 K for 1 h., a white precipitate appeared, which was washed with ethanol. The filtrates were combined and concentrated under reduced pressure to give a solid product, (2). Ethyl-triphenylphosphoranylidene (6.64 g, 19.06 mmol) was added to a solution of (2), and stirred for 2 h. at 298 K. The mixture was then extracted with CH_2_Cl_2_, in order to eliminate oxide triphenylphosphine, acidified until pH 3, and extracted again with ethyl acetate. The organic phase was dried over Na_2_SO_4_ and concentrated, to give (3) as a very thick syrup. By adding *N*,*N*'-dicyclohexylcarbodiimide (DCC) to a dry-CH_2_Cl_2_ solution of (3), under Ar, the lactone (I) was formed over 2 h. The solution was filtered in order to eliminate urea, and the filtrate concentrated, to give (I) as a white solid (3.58 g, 11.93 mmol; 73% yield). NMR and mass spectra are in agreement with the X-ray structure (see archived CIF). Single crystals were obtained by evaporation of an AcOEt solution of (I), at 298 K.

### Refinement

The absolute configuration was assigned after refining the Flack parameter (Flack, 1983), using 2010 measured Friedel pairs. All H atoms were placed in idealized positions, and refined as riding to their carrier atoms. C—H bond lengths were fixed to 0.93 (C*sp*^2^—H bonds) or 0.98 Å (methine CH groups), and isotropic displacement parameters calculated as *U*_iso_(H) = 1.2*U*_eq_(carrier C).

### Crystal data

**Table:**

C_9_H_7_Cl_3_O_5_	*D*_x_ = 1.682 Mg m^−^^3^
*M**_r_* = 301.50	Melting point = 416–418 K
Orthorhombic, *P*2_1_2_1_2_1_	Mo *K*α radiation λ = 0.71073 Å
Hall symbol: P 2ac 2ab	Cell parameters from 78 reflections
*a* = 9.129 (4) Å	θ = 4.6–12.4º
*b* = 11.264 (4) Å	µ = 0.77 mm^−^^1^
*c* = 23.156 (7) Å	*T* = 298 (1) K
*V* = 2381.1 (15) Å^3^	Cell measurement pressure: 101(2) kPa
*Z* = 8	Prism, colourless
*F*_000_ = 1216	0.60 × 0.40 × 0.10 mm

### Data collection

**Table:**

Siemens P4 diffractometer	*R*_int_ = 0.044
Radiation source: fine-focus sealed tube	θ_max_ = 26.2º
Monochromator: graphite	θ_min_ = 1.8º
*T* = 298(1) K	*h* = −11→11
ω scans	*k* = −14→14
Absorption correction: ψ scan(XSCANS; Siemens, 1996)	*l* = −28→28
*T*_min_ = 0.803, *T*_max_ = 0.926	3 standard reflections
6901 measured reflections	every 97 reflections
4735 independent reflections	intensity decay: 2%
3838 reflections with *I* > 2σ(*I*)	

### Refinement

**Table:**

Refinement on *F*^2^	Hydrogen site location: inferred from neighbouring sites
Least-squares matrix: full	H-atom parameters constrained
*R*[*F*^2^ > 2σ(*F*^2^)] = 0.044	*w* = 1/[σ^2^(*F*_o_^2^) + (0.0319*P*)^2^ + 1.7306*P*] where *P* = (*F*_o_^2^ + 2*F*_c_^2^)/3
*wR*(*F*^2^) = 0.111	(Δ/σ)_max_ < 0.001
*S* = 1.06	Δρ_max_ = 0.34 e Å^−^^3^
4735 reflections	Δρ_min_ = −0.35 e Å^−^^3^
307 parameters	Extinction correction: none
Primary atom site location: structure-invariant direct methods	Absolute structure: Flack (1983), 2010 Friedel pairs
Secondary atom site location: difference Fourier map	Flack parameter: 0.04 (8)

### Fractional atomic coordinates and isotropic or equivalent isotropic displacement parameters (Å^2^)

**Table:**

	*x*	*y*	*z*	*U*_iso_*/*U*_eq_	
Cl1	1.0538 (2)	0.40323 (18)	0.81819 (5)	0.1242 (7)	
Cl2	0.80275 (18)	0.39332 (15)	0.74470 (6)	0.1046 (5)	
Cl3	0.87777 (16)	0.60974 (15)	0.80223 (5)	0.0921 (4)	
O1	1.0741 (4)	0.3469 (3)	0.60422 (14)	0.0810 (10)	
C2	1.1264 (5)	0.4432 (4)	0.63415 (15)	0.0612 (10)	
H2A	1.2277	0.4611	0.6232	0.073*	
O2	1.1138 (3)	0.4251 (3)	0.69476 (11)	0.0730 (9)	
C3	1.0260 (4)	0.5478 (3)	0.62175 (13)	0.0510 (8)	
H3A	1.0796	0.6165	0.6067	0.061*	
O3	0.9574 (3)	0.5723 (2)	0.67532 (9)	0.0533 (6)	
C3A	0.9171 (4)	0.5005 (3)	0.57924 (14)	0.0486 (8)	
H3AA	0.8186	0.5317	0.5865	0.058*	
O4	0.9700 (3)	0.5335 (2)	0.52305 (9)	0.0540 (6)	
C5	0.9475 (5)	0.4644 (4)	0.47699 (16)	0.0626 (11)	
O5	0.9798 (5)	0.5044 (3)	0.43064 (11)	0.0863 (11)	
C6	0.8919 (7)	0.3451 (4)	0.4862 (2)	0.0852 (16)	
H6A	0.8621	0.3004	0.4546	0.102*	
C7	0.8828 (7)	0.2992 (4)	0.5377 (2)	0.0914 (18)	
H7A	0.8493	0.2218	0.5421	0.110*	
C7A	0.9250 (6)	0.3685 (4)	0.58927 (18)	0.0684 (12)	
H7AA	0.8616	0.3469	0.6218	0.082*	
C8	1.0435 (5)	0.5219 (4)	0.71861 (14)	0.0561 (10)	
H8A	1.1158	0.5798	0.7323	0.067*	
C9	0.9472 (5)	0.4813 (4)	0.76875 (16)	0.0675 (12)	
Cl11	0.48873 (14)	0.45925 (9)	0.55424 (4)	0.0678 (3)	
Cl12	0.32408 (13)	0.58949 (15)	0.47031 (5)	0.0852 (4)	
Cl13	0.63514 (12)	0.59974 (13)	0.46866 (4)	0.0733 (3)	
O11	0.3340 (3)	0.6649 (2)	0.68090 (10)	0.0568 (7)	
C12	0.3800 (5)	0.7463 (3)	0.63918 (14)	0.0527 (9)	
H12A	0.3338	0.8239	0.6452	0.063*	
O12	0.3488 (3)	0.7016 (3)	0.58318 (10)	0.0583 (7)	
C13	0.5434 (5)	0.7557 (3)	0.64229 (13)	0.0503 (9)	
H13A	0.5779	0.8380	0.6443	0.060*	
O13	0.5930 (3)	0.6951 (2)	0.59195 (10)	0.0506 (6)	
C13A	0.5833 (4)	0.6837 (3)	0.69550 (14)	0.0476 (8)	
H13B	0.6778	0.6433	0.6912	0.057*	
O14	0.5842 (3)	0.7680 (2)	0.74220 (10)	0.0552 (7)	
C15	0.5407 (5)	0.7344 (3)	0.79577 (14)	0.0547 (9)	
O15	0.5549 (4)	0.8058 (3)	0.83396 (11)	0.0814 (10)	
C16	0.4746 (5)	0.6189 (3)	0.80334 (14)	0.0598 (10)	
H16A	0.4626	0.5891	0.8405	0.072*	
C17	0.4312 (5)	0.5554 (3)	0.75938 (15)	0.0542 (9)	
H17A	0.3833	0.4836	0.7655	0.065*	
C17A	0.4586 (4)	0.5981 (3)	0.69935 (13)	0.0460 (8)	
H17B	0.4750	0.5307	0.6734	0.055*	
C18	0.4790 (4)	0.7002 (3)	0.55198 (14)	0.0498 (8)	
H18A	0.4872	0.7724	0.5286	0.060*	
C19	0.4815 (4)	0.5908 (3)	0.51320 (13)	0.0500 (8)	

### Atomic displacement parameters (Å^2^)

**Table:**

	*U*^11^	*U*^22^	*U*^33^	*U*^12^	*U*^13^	*U*^23^
Cl1	0.1478 (14)	0.1669 (16)	0.0579 (6)	0.0583 (13)	0.0186 (8)	0.0493 (9)
Cl2	0.1170 (11)	0.1059 (11)	0.0909 (9)	−0.0468 (9)	0.0423 (8)	−0.0014 (8)
Cl3	0.0981 (9)	0.1150 (11)	0.0632 (6)	0.0175 (8)	0.0090 (6)	−0.0219 (7)
O1	0.119 (3)	0.0595 (19)	0.0646 (18)	0.0256 (19)	0.0019 (19)	0.0040 (15)
C2	0.068 (2)	0.074 (3)	0.0413 (18)	0.009 (2)	0.0125 (18)	0.0106 (19)
O2	0.082 (2)	0.094 (2)	0.0429 (13)	0.0327 (18)	0.0134 (13)	0.0182 (15)
C3	0.059 (2)	0.0539 (19)	0.0401 (16)	−0.0027 (19)	0.0064 (16)	0.0059 (15)
O3	0.0669 (16)	0.0559 (14)	0.0371 (11)	0.0089 (13)	0.0027 (11)	0.0023 (10)
C3A	0.060 (2)	0.0480 (19)	0.0382 (16)	−0.0023 (17)	0.0064 (16)	0.0007 (15)
O4	0.0784 (17)	0.0454 (13)	0.0380 (11)	−0.0015 (13)	0.0041 (13)	0.0050 (10)
C5	0.089 (3)	0.055 (2)	0.0439 (19)	0.008 (2)	0.010 (2)	−0.0023 (17)
O5	0.141 (3)	0.0767 (19)	0.0410 (13)	0.003 (2)	0.0180 (18)	0.0018 (13)
C6	0.137 (5)	0.059 (3)	0.060 (3)	−0.015 (3)	0.002 (3)	−0.015 (2)
C7	0.153 (5)	0.054 (3)	0.067 (3)	−0.021 (3)	0.008 (3)	−0.004 (2)
C7A	0.106 (4)	0.051 (2)	0.048 (2)	−0.016 (2)	0.009 (2)	0.0023 (18)
C8	0.056 (2)	0.071 (3)	0.0408 (17)	0.005 (2)	0.0053 (17)	0.0085 (17)
C9	0.075 (3)	0.082 (3)	0.0449 (19)	0.009 (2)	0.0119 (19)	0.0139 (19)
Cl11	0.0934 (8)	0.0501 (5)	0.0599 (5)	−0.0081 (5)	0.0111 (6)	−0.0027 (4)
Cl12	0.0661 (6)	0.1375 (12)	0.0518 (5)	0.0059 (7)	−0.0119 (5)	−0.0211 (7)
Cl13	0.0659 (6)	0.1098 (9)	0.0443 (5)	−0.0028 (6)	0.0161 (4)	0.0017 (6)
O11	0.0608 (16)	0.0652 (17)	0.0443 (13)	0.0013 (13)	−0.0010 (12)	0.0127 (12)
C12	0.077 (3)	0.047 (2)	0.0342 (16)	0.0121 (19)	−0.0010 (17)	−0.0003 (15)
O12	0.0589 (16)	0.080 (2)	0.0364 (12)	0.0177 (14)	−0.0038 (11)	−0.0060 (12)
C13	0.075 (3)	0.0403 (17)	0.0355 (15)	−0.0042 (18)	0.0029 (16)	0.0013 (14)
O13	0.0557 (14)	0.0606 (16)	0.0356 (11)	−0.0098 (12)	0.0012 (10)	−0.0033 (11)
C13A	0.063 (2)	0.0419 (18)	0.0377 (16)	0.0000 (16)	−0.0030 (15)	−0.0069 (15)
O14	0.0838 (18)	0.0433 (13)	0.0386 (11)	−0.0123 (13)	−0.0030 (12)	−0.0040 (10)
C15	0.080 (3)	0.0457 (18)	0.0378 (16)	−0.0019 (19)	−0.0027 (18)	−0.0038 (15)
O15	0.137 (3)	0.0614 (17)	0.0461 (14)	−0.0131 (19)	−0.0012 (17)	−0.0150 (14)
C16	0.093 (3)	0.050 (2)	0.0359 (16)	0.001 (2)	−0.0011 (19)	0.0065 (15)
C17	0.080 (3)	0.0399 (18)	0.0430 (17)	−0.0034 (18)	−0.0025 (18)	0.0076 (15)
C17A	0.065 (2)	0.0376 (16)	0.0356 (14)	0.0000 (17)	−0.0054 (15)	−0.0001 (13)
C18	0.062 (2)	0.0500 (19)	0.0376 (15)	0.0018 (17)	0.0035 (17)	0.0075 (15)
C19	0.053 (2)	0.064 (2)	0.0326 (14)	−0.0013 (19)	0.0015 (14)	−0.0001 (15)

### Geometric parameters (Å, °)

**Table:**

Cl1—C9	1.741 (4)	Cl11—C19	1.761 (4)
Cl2—C9	1.741 (5)	Cl12—C19	1.747 (4)
Cl3—C9	1.760 (5)	Cl13—C19	1.744 (4)
O1—C2	1.373 (6)	O11—C12	1.397 (4)
O1—C7A	1.426 (6)	O11—C17A	1.429 (4)
C2—O2	1.423 (4)	C12—O12	1.420 (4)
C2—C3	1.520 (6)	C12—C13	1.497 (6)
C2—H2A	0.9800	C12—H12A	0.9800
O2—C8	1.381 (5)	O12—C18	1.390 (4)
C3—O3	1.417 (4)	C13—O13	1.424 (4)
C3—C3A	1.497 (5)	C13—C13A	1.520 (5)
C3—H3A	0.9800	C13—H13A	0.9800
O3—C8	1.395 (4)	O13—C18	1.394 (4)
C3A—O4	1.436 (4)	C13A—O14	1.439 (4)
C3A—C7A	1.507 (6)	C13A—C17A	1.495 (5)
C3A—H3AA	0.9800	C13A—H13B	0.9800
O4—C5	1.337 (5)	O14—C15	1.356 (4)
C5—O5	1.201 (5)	C15—O15	1.202 (4)
C5—C6	1.452 (7)	C15—C16	1.445 (5)
C6—C7	1.302 (6)	C16—C17	1.306 (5)
C6—H6A	0.9300	C16—H16A	0.9300
C7—C7A	1.478 (6)	C17—C17A	1.492 (5)
C7—H7A	0.9300	C17—H17A	0.9300
C7A—H7AA	0.9800	C17A—H17B	0.9800
C8—C9	1.527 (5)	C18—C19	1.526 (5)
C8—H8A	0.9800	C18—H18A	0.9800
			
C2—O1—C7A	108.6 (4)	C12—O11—C17A	108.3 (3)
O1—C2—O2	110.9 (4)	O11—C12—O12	109.8 (3)
O1—C2—C3	107.9 (3)	O11—C12—C13	108.2 (3)
O2—C2—C3	104.4 (3)	O12—C12—C13	105.6 (3)
O1—C2—H2A	111.1	O11—C12—H12A	111.0
O2—C2—H2A	111.1	O12—C12—H12A	111.0
C3—C2—H2A	111.1	C13—C12—H12A	111.0
C8—O2—C2	108.6 (3)	C18—O12—C12	107.9 (3)
O3—C3—C3A	110.6 (3)	O13—C13—C12	104.1 (3)
O3—C3—C2	104.6 (3)	O13—C13—C13A	109.4 (3)
C3A—C3—C2	104.4 (3)	C12—C13—C13A	103.9 (3)
O3—C3—H3A	112.3	O13—C13—H13A	112.9
C3A—C3—H3A	112.3	C12—C13—H13A	112.9
C2—C3—H3A	112.3	C13A—C13—H13A	112.9
C8—O3—C3	107.5 (3)	C18—O13—C13	106.6 (3)
O4—C3A—C3	106.3 (3)	O14—C13A—C17A	112.7 (3)
O4—C3A—C7A	112.3 (3)	O14—C13A—C13	105.0 (3)
C3—C3A—C7A	102.6 (3)	C17A—C13A—C13	102.1 (3)
O4—C3A—H3AA	111.7	O14—C13A—H13B	112.1
C3—C3A—H3AA	111.7	C17A—C13A—H13B	112.1
C7A—C3A—H3AA	111.7	C13—C13A—H13B	112.1
C5—O4—C3A	121.4 (3)	C15—O14—C13A	120.1 (3)
O5—C5—O4	117.2 (4)	O15—C15—O14	117.0 (3)
O5—C5—C6	124.4 (4)	O15—C15—C16	123.9 (3)
O4—C5—C6	118.4 (3)	O14—C15—C16	119.0 (3)
C7—C6—C5	121.6 (4)	C17—C16—C15	121.7 (3)
C7—C6—H6A	119.2	C17—C16—H16A	119.1
C5—C6—H6A	119.2	C15—C16—H16A	119.1
C6—C7—C7A	120.9 (4)	C16—C17—C17A	119.9 (3)
C6—C7—H7A	119.5	C16—C17—H17A	120.0
C7A—C7—H7A	119.5	C17A—C17—H17A	120.0
O1—C7A—C7	110.8 (5)	O11—C17A—C17	108.4 (3)
O1—C7A—C3A	104.6 (4)	O11—C17A—C13A	104.4 (3)
C7—C7A—C3A	112.6 (4)	C17—C17A—C13A	113.0 (3)
O1—C7A—H7AA	109.6	O11—C17A—H17B	110.3
C7—C7A—H7AA	109.6	C17—C17A—H17B	110.3
C3A—C7A—H7AA	109.6	C13A—C17A—H17B	110.3
O2—C8—O3	107.2 (3)	O12—C18—O13	107.1 (2)
O2—C8—C9	109.6 (3)	O12—C18—C19	109.1 (3)
O3—C8—C9	110.1 (3)	O13—C18—C19	110.3 (3)
O2—C8—H8A	110.0	O12—C18—H18A	110.1
O3—C8—H8A	110.0	O13—C18—H18A	110.1
C9—C8—H8A	110.0	C19—C18—H18A	110.1
C8—C9—Cl1	109.3 (3)	C18—C19—Cl13	108.3 (3)
C8—C9—Cl2	111.3 (3)	C18—C19—Cl12	109.2 (3)
Cl1—C9—Cl2	110.3 (3)	Cl13—C19—Cl12	109.01 (16)
C8—C9—Cl3	107.2 (3)	C18—C19—Cl11	111.3 (2)
Cl1—C9—Cl3	109.1 (2)	Cl13—C19—Cl11	109.7 (2)
Cl2—C9—Cl3	109.6 (3)	Cl12—C19—Cl11	109.3 (2)
			
C7A—O1—C2—O2	94.8 (4)	C17A—O11—C12—O12	99.0 (3)
C7A—O1—C2—C3	−19.0 (4)	C17A—O11—C12—C13	−15.8 (4)
O1—C2—O2—C8	−128.5 (4)	O11—C12—O12—C18	−124.3 (3)
C3—C2—O2—C8	−12.5 (5)	C13—C12—O12—C18	−7.8 (4)
O1—C2—C3—O3	113.3 (3)	O11—C12—C13—O13	107.1 (3)
O2—C2—C3—O3	−4.7 (4)	O12—C12—C13—O13	−10.4 (4)
O1—C2—C3—C3A	−2.9 (4)	O11—C12—C13—C13A	−7.4 (4)
O2—C2—C3—C3A	−120.9 (3)	O12—C12—C13—C13A	−124.9 (3)
C3A—C3—O3—C8	132.0 (3)	C12—C13—O13—C18	25.0 (4)
C2—C3—O3—C8	20.1 (4)	C13A—C13—O13—C18	135.5 (3)
O3—C3—C3A—O4	151.9 (3)	O13—C13—C13A—O14	157.7 (3)
C2—C3—C3A—O4	−96.1 (3)	C12—C13—C13A—O14	−91.6 (3)
O3—C3—C3A—C7A	−90.1 (4)	O13—C13—C13A—C17A	−84.5 (3)
C2—C3—C3A—C7A	21.9 (4)	C12—C13—C13A—C17A	26.1 (3)
C3—C3A—O4—C5	147.0 (4)	C17A—C13A—O14—C15	35.4 (5)
C7A—C3A—O4—C5	35.6 (5)	C13—C13A—O14—C15	145.7 (3)
C3A—O4—C5—O5	171.5 (4)	C13A—O14—C15—O15	174.2 (4)
C3A—O4—C5—C6	−10.9 (6)	C13A—O14—C15—C16	−8.6 (6)
O5—C5—C6—C7	167.8 (6)	O15—C15—C16—C17	164.2 (5)
O4—C5—C6—C7	−9.5 (8)	O14—C15—C16—C17	−12.7 (7)
C5—C6—C7—C7A	1.8 (10)	C15—C16—C17—C17A	4.1 (7)
C2—O1—C7A—C7	154.9 (4)	C12—O11—C17A—C17	153.7 (3)
C2—O1—C7A—C3A	33.4 (4)	C12—O11—C17A—C13A	33.0 (3)
C6—C7—C7A—O1	−93.4 (7)	C16—C17—C17A—O11	−92.0 (5)
C6—C7—C7A—C3A	23.4 (8)	C16—C17—C17A—C13A	23.2 (5)
O4—C3A—C7A—O1	80.2 (4)	O14—C13A—C17A—O11	76.1 (3)
C3—C3A—C7A—O1	−33.5 (4)	C13—C13A—C17A—O11	−35.9 (3)
O4—C3A—C7A—C7	−40.1 (6)	O14—C13A—C17A—C17	−41.4 (4)
C3—C3A—C7A—C7	−153.8 (4)	C13—C13A—C17A—C17	−153.5 (3)
C2—O2—C8—O3	25.7 (4)	C12—O12—C18—O13	23.9 (4)
C2—O2—C8—C9	145.1 (4)	C12—O12—C18—C19	143.2 (3)
C3—O3—C8—O2	−28.8 (4)	C13—O13—C18—O12	−30.9 (3)
C3—O3—C8—C9	−147.9 (3)	C13—O13—C18—C19	−149.5 (3)
O2—C8—C9—Cl1	55.3 (4)	O12—C18—C19—Cl13	173.3 (2)
O3—C8—C9—Cl1	173.0 (3)	O13—C18—C19—Cl13	−69.4 (3)
O2—C8—C9—Cl2	−66.7 (4)	O12—C18—C19—Cl12	54.7 (3)
O3—C8—C9—Cl2	51.0 (4)	O13—C18—C19—Cl12	172.1 (2)
O2—C8—C9—Cl3	173.4 (3)	O12—C18—C19—Cl11	−66.0 (3)
O3—C8—C9—Cl3	−68.9 (4)	O13—C18—C19—Cl11	51.3 (4)
